# Telemedicine-based serious illness conversations, healthcare utilization, and end of life care among patients with advanced lung cancer

**DOI:** 10.1093/oncolo/oyae216

**Published:** 2024-08-29

**Authors:** Tejaswini M Dhawale, Roopa S Bhat, P Connor Johnson, Shanivi Srikonda, Kelsey S Lau-Min, Kofi Boateng, Howard Lee, Hermioni L Amonoo, Ryan Nipp, Charlotta Lindvall, Areej El-Jawahri

**Affiliations:** Department of Medicine, Division of Hematology and Oncology, Massachusetts General Hospital, Boston, MA, United States; Harvard Medical School, Boston, MA, United States; University of Colorado School of Medicine, Aurora, CO, United States; Department of Medicine, Division of Hematology and Oncology, Massachusetts General Hospital, Boston, MA, United States; Harvard Medical School, Boston, MA, United States; Harvard University, Boston, MA, United States; Department of Medicine, Division of Hematology and Oncology, Massachusetts General Hospital, Boston, MA, United States; Harvard Medical School, Boston, MA, United States; Department of Medicine, Division of Hematology and Oncology, Massachusetts General Hospital, Boston, MA, United States; Division of Hematology &Oncology, University of California San Francisco, San Francisco, CA, United States; Harvard Medical School, Boston, MA, United States; Department of Psychiatry, Brigham and Women’s Hospital, Boston, MA, United States; Department of Psychosocial Oncology and Palliative Care, Dana-Farber Cancer Institute, Boston, MA, United States; Section of Hematology/Oncology, Department of Internal Medicine, University of Oklahoma Health Sciences Center, Stephenson Cancer Center, Oklahoma City, OK, United States; Harvard Medical School, Boston, MA, United States; Clinical Informatics, Dana Farber Cancer Institute, Boston, MA, United States; Department of Medicine, Division of Hematology and Oncology, Massachusetts General Hospital, Boston, MA, United States; Harvard Medical School, Boston, MA, United States

## Abstract

**Purpose:**

Little is known about serious illness conversations (*SIC*) conducted during telemedicine visits and their impact on end-of-life (EOL) outcomes for patients with advanced cancer.

**Patients and Methods:**

We conducted a retrospective analysis telemedicine visits for patients with metastatic lung cancer conducted during the first surge of the COVID-19 pandemic (October 3, 2020-October 6, 2020). We used natural language processing (NLP) to characterize documentation of *SIC* domains (ie, goals of care [GOC], limitation of life-sustaining treatment [LLST], prognostic awareness [PA], palliative care [PC], and hospice). We used unadjusted logistic regression to evaluate factors associated with SIC documentation and the relationship between SIC documentation and EOL outcomes.

**Results:**

The study included 634 telemedicine visits across 360 patients. Documentation of at least one SIC domain was present in 188 (29.7%) visits with GOC and PA being the most discussed domains. Family presence (odds ratio [OR], 1.66; *P* = .004), progressive or newly diagnosed disease (OR, 5.42; *P* < .000), age ≥ 70 (OR, 1.80; *P* = .009), and male sex (OR, 2.23; *P* < .000) were associated with a greater likelihood of discussing ≥ 1 *SIC* domain. Of the 61 patients who died within 12 months of the study period, having ≥ 1 *SIC* domain discussed was associated with a lower likelihood of hospitalization in the last 30 days of life (OR, 0.27; *P* = .020).

**Conclusion:**

In this study of telehealth visits, we identified important factors associated with an increased likelihood of having documentation of an *SIC* and demonstrated that *SIC* documentation correlated with lower likelihood of hospitalization at EOL.

## Teaser

This article describes the frequency, content, and contextual circumstances of serious illness conversations conducted during telemedicine visits of patients with metastatic lung cancer during the COVID-19 pandemic and explores end-of-life outcomes.

**Implications for practice**

In this study, we identified factors (age, disease status, and family presence) associated with an increased likelihood of having a serious illness conversation (*SIC*) during telemedicine visits and found that clinicians most commonly address goals of care and prognostic awareness during these discussions. Importantly, we found that documentation of an *SIC* during a telemedicine encounter was associated with a lower likelihood of hospitalization in the last 30 days of life for patients with metastatic lung cancer. Collectively, our findings provide novel insight on the feasibility of telemedicine-based SICS and their potential to improve EOL cancer care.

## Introduction

Serious illness conversations (SIC) are discussions that explore patients’ understanding, goals, and preferences about their care.^1^ For patients with cancer, SICs encompass a range of topics including prognosis, goals of care (GOC), treatment decisions, advanced care planning (ACP), and preferences for end-of-life (EOL) care.^2^ SICs improve EOL outcomes and are associated with improved quality of life, less intensive medical care at the EOL, and earlier referrals to hospice services in oncology populations.^3,4^ The COVID-19 pandemic highlighted the value of SICs for oncology patients, particularly during the initial phase when hospitals experienced shortages of healthcare resources.^5,6^ Many patients with advanced cancer faced difficult decisions about pursuing cancer treatment with the added risk of COVID-19 infection and experiencing potentially life-threatening illness.^5,7^ In response, healthcare systems encouraged oncologists to use telemedicine for *SIC* discussions, often with limited training and few metrics of patient or clinician satisfaction.^8^ To date, very little is known about the content of SICs using telemedicine and the impact of these conversations on long-term EOL outcomes for patients with advanced cancer. A comprehensive understanding of SICs for patients with advanced cancer conducted via telemedicine will clarify how clinicians can leverage virtual technologies for patient-centered care.

In the present study, we aimed to describe the frequency, content, and contextual circumstances of SICs conducted during telemedicine visits of patients with metastatic lung cancer during the COVID-19 pandemic. We also explored patient factors associated with *SIC* and the relationship between *SIC* and EOL outcomes. Enhancing our understanding of SICs conducted using telemedicine will provide novel insights to address current gaps in EOL care and inform interventions to improve SICs.

## Methods

### Study Design and Setting

We conducted a retrospective study of telemedicine visit data from the electronic health records (EHRs) of adult (age ≥ 18) patients with metastatic lung cancer during the first surge of COVID-19 (October 3, 2020-October 6, 2020) at the Massachusetts General Hospital (MGH) Cancer Center. During this time, outpatient clinic visits were severely restricted and oncology clinicians were encouraged to conduct patient encounters using telemedicine given unprecedented shortages in critical care resources at that time and the need to minimize patient exposure to COVID19 from in-person consultations. This study was reviewed and approved by the Dana-Farber/Harvard Cancer Center Institutional Review Board.

### Data Collection

All data were obtained from the MGH Research Patient Data Registry (RPDR) database, our institution’s centralized clinical data registry, our EHR (Epic Systems, Inc.), and other administrative resources.

#### Participants

We identified all patients with metastatic (stage IV) lung cancer who had telemedicine visits with a provider (oncologist or advanced practice provider) at the MGH Thoracic Oncology clinic during the study period. We focused on patients with metastatic non-small cell or small cell lung cancer given their baseline poor prognosis, heightened risk of respiratory decompensation, and need for critical care resources of these patients with COVID-19 infection during the study period.

#### Participant and Encounter Characteristics

Sociodemographic information of all patients and date of death were obtained from RPDR and confirmed by manual chart review. Telemedicine encounter characteristics (date, video vs phone, physician vs advanced practice provider) were obtained from our administrative database.

### SIC Documentation Review Methodology

All plain-text notes from telemedicine visits were extracted from the EHR using the RPDR database.

#### Abstraction Protocol Overview

We utilized a previously validated natural language processing (NLP)-based algorithm to facilitate identification of SICs in the EHR.^9^ We expanded the previously published abstraction protocol by adding contextual domains (described below) and an *SIC* domain for prognostic awareness (PA). The final abstraction protocol defined *SIC* documentation as a provider note documenting one or more of five *SIC* domains: (1) GOC, ie, documentation about values, goals, priorities for treatment and outcomes; (2) limitation of life-sustaining treatment [LLST], ie, documentation of preferences about cardiopulmonary resuscitation or intubation; (3) PA, ie, documentation about estimated chances of recovery or disease course; (4) palliative care [PC], ie, documentation of PC consultation or referral]; and (5) hospice ie, documentation of hospice referral or patient enrollment preferences (Supplemental Appendix 1). Documentation from each telemedicine encounter was scored with a “0” (absent) or “1” (present) for each domain. The abstraction protocol also included categories that captured contextual elements of the conversations including (1) the presence or absence of family during the encounter and (2) the status of the patient’s cancer at the time of the encounter.

### Data Extraction

We used NLP text annotation software (Clinical Regex, Lindvall Lab)^8,9^ to identify and characterize *SIC* discussions. Clinical Regex employs a pre-defined ontology to highlight keywords or phrases of interest within clinical notes. This NLP approach enables a semi-automated chart review, facilitating rapid extraction of key data from text-based clinical notes. Our ontology for identifying documentation of SICs utilized a previously published keyword library with categories for four *SIC* domains (GOC, LLST, hospice, and PC).^9^ We modified this keyword library using an iterative process to create a final library that was consistent with the abstraction protocol. Major modifications included adding the categories “PA” to the *SIC* domains, and “family/proxy” and “disease status” to the contextual domains as noted before. This *SIC* NLP methodology has been used and validated in prior studies.^9^

Assisted by NLP, 2 coders (T.D., R.B.) reviewed the telemedicine visit notes and recorded the presence or absence of documentation of at least one *SIC* domain, which *SIC* domains were discussed, whether family was present and the status of the patient’s cancer at the time of the visit. We assessed interrater reliability on a subset of 113 (18%) telemedicine notes which were independently abstracted and double coded for the presence or absence of *SIC* using Cohen’s kappa. There was substantial agreement between coders, κ = 0.70 (95% CI, 0.567-0.833), *P* < .001.

#### EOL Outcomes

The EOL outcomes were selected based on established health services quality metrics.^10-13^ To collect data on EOL outcomes, we conducted a comprehensive chart review on all patients who died in the 12 months following the start of the study period (October 3, 2020 to October 3, 2021). We abstracted information about the presence or absence of ACP documentation in the EHR, dates of referrals to hospice, the presence of referrals to PC services, location of death, and use of chemotherapy in the last 14 days of life. For healthcare utilization, we reviewed the frequency and dates of hospitalizations, isolated emergency department (ED) visits (without hospital admission), and intensive care unit (ICU) admissions.

### Statistical Analysis

We used descriptive statistics to summarize patients’ demographic and clinical characteristics, telemedicine encounter characteristics, documentation of *SIC* domains, health care utilization, and EOL outcomes.

We then examined factors associated with documentation of at least one *SIC* domain (yes vs no) using unadjusted logistic regression analysis. We specifically investigated the association of *SIC* documentation and patient demographic (ie, age ≥ 70 [analyzed as a binary variable], gender, race, relationship status, primary language), cancer status (cancer stable or in remission vs progressing or new diagnosis), encounter characteristics (clinician type [MD vs advanced practice provider], encounter modality [phone vs video], and family attendance [absent vs present]) with the binary outcome of interest. These factors were selected a priori based on prior research indicating that these covariates correlate with ACP and GOC conversations.^14-20^

We also used unadjusted logistic regression models to investigate the association of documentation of at least one *SIC* domain (yes vs no) with hospice utilization (hospice referral [yes vs no], hospice length of stay (LOS) [dichotomized to 0-7 days vs > 7 days]) and binary (yes vs no) EOL quality outcomes (chemotherapy in the last 14 days of life, ICU utilization in the last 30 days of life, ED utilization in the last 30 days of life, and hospitalization in the last 30 days of life). Due to relatively small sample size and low event rates for these outcomes, we were unable to conduct multivariable analyses. All reported *P*-values were 2-sided with a *P*-value < .05 considered statistically significant. We conducted statistical analyses using STATA, version 17.0 (StataCorp LLP).

## Results

### Clinician and Patient Characteristics

We identified 360 patients with metastatic lung cancer, seen by 17 oncology clinicians via telemedicine, with 634 total visits throughout the study period. Among all patients, the mean age was 66 (range 32-93). The majority of patients were female (214, 59.4%), white race (298, 82.8%), married or living with a partner (256, 71.1%), English-speaking (330, 81.7%), and had a diagnosis of non-small cell lung cancer (333, 92.5%; Table 1).

**Table 1.** Patient characteristics.

Characteristics	Mean (range) or *N* (%) *N* = 360
Age, mean (range), years	66 (32-93)
Sex: male	146 (40.6%)
Race	
White	298 (82.8%)
Asian	36 (10.0%)
African American	8 (2.2%)
American Indian or Alaska Native	1 (0.3%)
Other	9 (2.5%)
Missing/not reported	8 (2.2%)
Cancer	
Non-small cell lung cancer	333 (92.5%)
Small cell lung cancer (includes mixed histology)	27 (7.5%)
Relationship status	
Married/living with a partner	256(71.1%)
Single	44 (12.2%)
Divorced or legally separated	27 (7.5%)
Widowed	30 (8.3%)
Other	2 (0.6%)
Missing/not reported	1 (0.3%)
Self-reported language preference	
English	330 (91.7%)
Non-English	30 (8.3%)

### SIC Documentation During Telemedicine Visits

Of the 634 telemedicine visits identified, 556 (87.7%) were visits with a physician (vs advanced practice provider), and 314 (49.5%) were video based (vs telephone). We reviewed each encounter and found that 194 (30.6%) notes documented discussion about disease progression or new diagnosis of metastatic lung cancer, 248 (39.1%) documented discussion with one or more family members, and 188 (29.7%) documented addressing at least one *SIC* domain during the encounter (Table 2).

**Table 2.** Telemedicine encounter characteristics.

Characteristics	*N* = (%) Total: 634
Clinician type	
Physician	556 (87.7%)
Advanced practice provider	78 (12.3 %)
Visit medium	
Video	314 (49.5%)
Phone	320 (50.5%)
Cancer status at the time of visit	
Disease progression or new diagnosis	194 (30.6%)
Stable or no evidence of disease	364 (57.4%)
Unable to determine	76 (12.0)
Family status	
Family present with patient at the visit	248 (39.1%)
Family absent	380 (59.9%)
Only family present (patient absent)	6 (0.95%)
Number of *SIC* domains documented	
None	446 (70.3%)
1	110 (17.4%)
2 or more	78 (12.3%)

**Figure 1.**
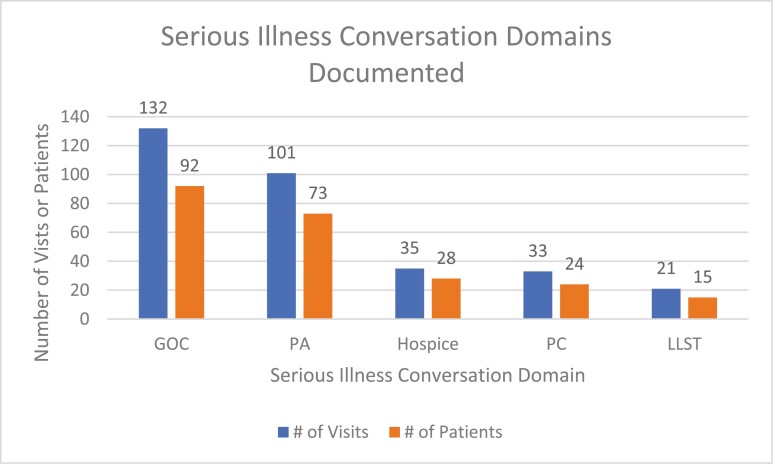
Number of visits and patients with documented *SIC* domains. Abbreviations: *SIC*, serious illness conversation, LLST, limits of life- sustaining treatment; PC, palliative care; PA, prognostic awareness; GOC, goals of care. #, number.

Of the 188 *SIC*-positive telemedicine visits (126 patients), 132 (70.2%) documented discussion of GOC, 101 (53.7%) addressed PA, 35 (18.6%) addressed hospice, 33 (17.6%) addressed PC, and 21 (11.2%) addressed limits of life sustaining therapy (Figure 1).

### Healthcare Utilization and EOL Outcomes

A total of 61 (16.9%) patients died in the 12 months following the start of the study period. Overall, 41 (67.2%) deceased patients were hospitalized, 18 (29.5%) were evaluated in the ED (without admission), and 13 (21.3%) were admitted to the ICU (Table 3). At the time of death, 51 (83.6%) had hospice referrals, 54 (88.5%) had PC referrals, and 47 (77.0%) had ACP documentation available in the EHR. While 37 (60.6%) died in hospice, 17 (27.9%) died in the hospital, with 5 deaths occurring in the ICU. Of patients referred to hospice, 11 (18.0%) were referred within the last 7 days of life.

**Table 3.** Healthcare utilization and end of life outcomes of deceased patients at 12 months.

Outcome	12 months^*^ (*N* = 61)
Hospice utilization	
Number referred (%)	51 (83.6%)
Hospice LOS < 7 days (% of those referred)	11 (18.0%)
Palliative care (%) utilization	
Number referred (%)	54 (88.5%)
ACP	
HCP identified (%)	47 (77.0%)
MOLST form (%)	30 (49.2%)
DNR code status (%)	43 (70.5%)
Location of death	
Hospice (at home or non-hospital facility)	37 (60.6%)
Hospital (including inpatient hospice)	17 (27.9%)
ICU (% of those who died in hospital)	5 (30%)
Unknown	7 (11.6%)
EOL quality outcomes	
Chemotherapy in the last 14 days of life	4 (6.6%)
Hospitalization in the last 30 days of life	22 (36.1%)
ED utilization (without admission) in the last 30 days of life	9 (14.8%)
ICU utilization in the last 30 days of life	9 (14.8%)
Healthcare utilization^*^	
Hospital admissions	41 (67.2%)
ED visits	18 (29.5%)
ICU admissions	13 (21.3%)

^*^Between October 3, 2020 and November 3, 2021.

In the last 30 days of life, 22 (36.1%) decedents were hospitalized, 9 (14.8%) were evaluated in the ED (without admission), and 9 (14.8%) were admitted to the ICU. In the last 14 days of life, 4 (6.6%) decedents received chemotherapy.

### Factors Associated with Documentation of *SIC* Domains

Family presence at the encounter (odds ratio [OR], 1.66; 95% CI, 1.17-2.34; *P* = .004), progressive or newly diagnosed disease (OR, 5.42; 95% CI, 3.68-8.01; *P *< .000), age ≥ 70 (OR, 1.80; 95% CI, 1.16-2.79; *P *= .009), and male sex (OR, 2.23; 95% CI, 1.43-3.47; *P* < .000) were associated with a greater likelihood of at least one *SIC* domain discussion (Table 4). Clinician type, encounter medium, race, relationship status, and primary language were not associated with likelihood of having at least one *SIC* domain discussion.

**Table 4.** Univariate logistic regression on factors associated with discussion of at least one *SIC* domain during telehealth visits.

Variables	OR	95% CI	*P*-value
Encounter characteristics			
Clinician type (ref. Physician)			
Advanced practice provider	0.63	0.36, 1.11	.107
Encounter medium (ref. phone)			
Video	0.84	0.59, 1.18	.306
Patient variables			
Age (ref. Age < 70)			
Age ≥ 70	1.80	1.16, 2.79	.009
Sex (ref. female)			
Male	2.23	1.43, 3.47	<.001
Race (ref. White)			
Non-white race	0.86	0.44,1.68	.649
Relationship status (ref. unmarried)			
Married or partnered	0.64	0.40, 1.03	.065
Language preference (ref. non-English)			
English	0.84	0.43, 2.00	.842
Contextual variables			
Cancer status (ref. stable or no evidence of disease)			<.001
Progressing or new diagnosis	5.42	3.68, 8.01	<.001
Family attendance (ref. absent)			
Family present with patient	1.66	1.17, 2.34	.004

Among patients who died within 12 months following the start of the study period, we found documentation of at least one *SIC* domain was associated with lower likelihood of hospitalization in the last 30 days of life (OR, 0.27; 95% CI, 0.09-0.82; *P *= .020; Table 5). Patients with documentation of at least one *SIC* domain also had a trend toward lower likelihood of ED and ICU utilization compared with those patients without *SIC* documentation (*P* = .082). Documentation of at least one *SIC* domain was not associated with likelihood of chemotherapy use within the last 2 weeks of life, hospice referral, or hospice LOS.

**Table 5.** Univariate logistic regression to identify the association of having at least one *SIC* domain addressed during a telemedicine encounter with EOL quality outcomes.

Variables	OR	95% CI	*P*-value
Hospice utilization (ref. no)			
Hospice referral (yes)	1.03	0.26, 4.13	.963
Hospice LOS < 7 days (yes)	1.17	0.30, 4.51	.823
EOL quality outcomes (ref. no)			
Chemotherapy in the last 14 days of life (yes)	0.63	0.08, 4.79	.654
ICU utilization in the last 30 days of life (yes)	0.26	0.06, 1.19	.082
ED utilization in the last 30 days of life (yes)	0.26	0.06, 1.19	.082
Hospitalization in the last 30 days of life (yes)	0.27	0.09, 0.82	.020

## Discussion

In this retrospective study using NLP to describe documentation of *SIC* in the EHR, we found that approximately one-third of patients who had telemedicine visits with their clinicians during the first surge of the COVID-19 pandemic had documentation of an *SIC*. During these SICs, clinicians most frequently discussed the domains of GOC and PA but rarely addressed the topics of hospice, PC, or LLST. We also identified factors associated with documentation of at least one *SIC* domain, such as the presence of family during the visit, new/worsening disease status, older age and male gender. Finally, we demonstrated that more than one-third of patients who died within a year of the study period were hospitalized in the last 30 days of life and that documentation of an *SIC* during a telemedicine encounter was associated with a lower likelihood of experiencing this adverse event. Taken together, our results demonstrate that telemedicine-based SICs are not only feasible but also correspond with more favorable EOL outcomes for patients with advanced lung cancer.

In this study, we observed that only a minority (one-third) of patients who had a telemedicine encounter during the study period had any documentation of an *SIC*. Moreover, the majority of the SICs identified in this study addressed only one *SIC* domain, suggesting that there is an underutilization of telemedicine visits to conduct SICs and potential to have more-in depth conversations. Compared to the observed frequency of telemedicine-based SICs in this study, Patel et al observed substantially lower rates of SICs during in-person visits with patients with high-risk cancer (3.4%), even after instituting a program of clinician prompts to encourage SICs (13.5%).^21^ One key reason for the difference may be that their study was conducted just before the onset of the COVID-19 pandemic. Another study conducted by Bernacki et al during the pandemic evaluated the quality of SICs conducted during in-person visits at that time and, similar to our study, found that most clinicians focused on discussions of values and goals followed by prognosis and illness understanding.^22^ Additional studies are necessary to further understand how the medium of these encounters influences the conduct and comprehensiveness of SICs.^23^

Our study is among the first studies to identify an association between specific contextual factors and the occurrence of an SIC during a telemedicine visit. We found that clinicians were more likely to conduct and document an SIC early in the disease trajectory (eg, at diagnosis), at the time of disease progression, in the presence of family during a telemedicine encounter, with older patients, and with male patients. These observations may be key to understanding the positive impact of telemedicine-based SICs on EOL outcomes. Indeed, we surmise that by fostering participation of family caregivers in discussions about the patient’s care, telemedicine facilitates surrogate decision makers’ understanding of the patients’ wishes and may stimulate further discussion between patients and their health care proxy about preferences for care.^24^ Prior work suggests that, cancer caregivers often have misperceptions about the patients’ likelihood of cure, as well as discordant perceptions from the patient.^25^ Plausibly, the positive effects of telemedicine-based SICs on healthcare utilization at the EOL could be mediated by improved caregiver understanding of the patient’s prognosis and GOC. Previous research has also demonstrated that advanced care planning correlates with decreased in-hospital death and increased use of hospice.^26^ Thus, telemedicine-based SICs may foster completion of ACP by patients by enabling involvement of caregivers in the planning process. While telemedicine offers the advantage of “delivering bad news” to patients in the comforts of a familiar environment and family, a dearth of research has sought to understand patient satisfaction with receiving difficult news via telemedicine. Future studies should explore patient satisfaction with telemedicine-based SICS as well as the impact of interventions that promote telemedicine-based *SIC* in different contexts.

Interestingly, while more than three-quarters of decedents in this study had some form of ACP documentation at the time of death, we found that only a minority of telemedicine-based SICs in our study involved ACP discussions. This discordance may be explained by the logistical encumbrances providers face when attempting to complete ACP documentations via telemedicine. In contrast to in-person visits, telemedicine visits do not lend themselves easily to discussions about ACP documents given the requirement for wet signatures from multiple parties including a live witness to complete many of these documents. In fact, before the pandemic, remote execution of advanced directives was only possible in 18 states, of which only 6 had legislation recognizing online execution of advanced directives (ie, using digital signatures, e-notarization, witness via audiovisual means, etc.).^27^ While the COVID-19 pandemic has forced many states to adopt new remote notarization and witnessing laws, many states still lag behind in taking the necessary legislative action to ensure that advanced directives can be remotely executed.^27^ Thus, while telemedicine has the potential to bridge significant communication gaps regarding ACP and thus reduce low quality care at the EOL, the full potential of telemedicine-based SICs may not be realized without the support of policymakers vested in making remote execution of advanced directives accessible to all people.

In this study, we found no significant association between encounter medium (ie, phone or video) and the occurrence of SICs. A recent study of Medicare beneficiaries evaluated patient preferences regarding telephone versus video visits and found that patients often preferred phone visits even when video visits were available.^28^ Patients were more likely to choose phone visits if they had limited access to technology, were in a lower socioeconomic group, and older (age 75-84).^28^ A second qualitative study of a telemedicine-based Serious Illness Care Program (SICP) delivered to older patients with hematologic malignancy found that older patients with limited experience using technology were willing and able to learn in order to engage with their providers. Importantly, in our study, older patients (age ≥ 70) had a higher likelihood of having documentation of at least one *SIC* domain during a telemedicine encounter. This suggests that phone and video visits may be equally effective in conducting SICs, and that older patients are more likely to participate in SICs using telemedicine. However, to achieve widespread use of telemedicine to conduct SICs, digital health care disparities that limit access to telemedicine in older and lower socioeconomic status populations must be first addressed. Therefore, future research should focus on investigating telemedicine-based SICs for scalability, promoting equity and improving access to care.^29^

At present, there have been no randomized controlled studies that have directly compared in-person SICs to telemedicine-based SICs. Previous investigations of in-person SICs identified increased burden of acute or chronic illness and older age as predictors of EHR-documented goals-of care discussions among hospitalized patients.^30^ In contrast to prior studies, we found that male gender was associated with a greater likelihood of at least one *SIC* domain discussion during a telemedicine-encounter. This is a striking difference from the literature given that multiple studies of in-person SICs have demonstrated that documented goals-of-care discussions are more common for women.^30,31^ This finding may reflect the increased comfort that men may feel in discussing their concerns in the less “intimate” or more emotionally distanced medium of telemedicine. It is also possible that these findings are influenced by limited sample size of the study population, and thus should be interpreted with caution. Additional research is necessary to determine the extent to which the medium of telehealth affects the conduct and content of SICs.

There are additional important limitations to this study. First, we conducted this study among patients who received care at a single large academic center that serves a predominantly White population, thus our findings may not generalize to other settings or populations. Second, the retrospective, observational design of the study dictates that we can only demonstrate associations and not causality. With limitations related to a relatively small sample size, we could not account for potential confounders, such as performance status, treatment regimen, or comorbid conditions that may have influenced the association between SICs and the EOL outcomes described in this study. Further, the data abstraction in this study was reliant on (1) an NLP-driven word search algorithm based on a pre-defined keyword library, (2) accuracy of the EHR, and (3) completeness of the medical record. It is possible that we may have overlooked some SICs if the words used by clinicians to conduct SICs did not match any of the words in the keyword library or if the clinician did not document their *SIC* with the patient. The method used in this study for data abstraction would also not capture any non-verbal communication elements of SICs. Additionally, clinicians often copy-and-paste documentation of prior visits into their notes and sometimes overlook the process of removing content that was not directly addressed during the current encounter. As such, our observed frequencies of SICs may have been inflated by errors due to this redundancy in the medical record. Conversely, we observed significant heterogeneity among providers regarding documentation of SICs given the lack of standardization in the documentation process of SICs. Consequently, the observed frequency of *SIC* domains may be lower than actual frequency given that the medical record might not reflect the full extent of SICs conducted during the encounter. Our data abstraction was limited to data existing in our EHR, so SICs that were conducted or documented outside of MGH or outcomes (hospitalizations, ER visits) that occurred in hospital systems not connected to the MGH EHR were not accounted for in this study.

## Conclusion

In summary, we identified factors (age, disease status, and family presence) associated with an increased likelihood of having an *SIC* during telemedicine visits and found that clinicians most commonly address GOC and PA during these discussions. We also demonstrated that documentation of an *SIC* during a telemedicine encounter was associated with a lower likelihood of hospitalization in the last 30 days of life for patients with metastatic lung cancer. Collectively, our findings provide novel insight on the feasibility of telemedicine-based SICS and their potential to improve EOL cancer care. Future research should focus on minimizing barriers and improving patient access to telemedicine-based SICs while also seeking to further understanding the potential impact of these vital conversations on cancer care delivery.

## Supplementary material

Supplementary material is available at *The Oncologist* online.

oyae216_suppl_Supplementary_Material

## Data Availability

The data underlying this article cannot be shared publicly due for the privacy of individuals that participated in the study. The data will be shared on reasonable request to the corresponding author.
