# Supplementary material for: Telemedicine-based serious illness conversations, healthcare utilization, and end of life care among patients with advanced lung cancer
Source: Oncologist. 2024 Aug 29;29(12):e1762–9. doi: 10.1093/oncolo/oyae216 (PMC11630782; doi:10.1093/oncolo/oyae216)
Supplement: oyae216_suppl_Supplementary_Material [file oyae216_suppl_supplementary_material.docx]

**Supplementary Appendix.**

**Table 1. Abstraction protocol for identifying Serious Illness Conversations During Telehealth Visits**

In order to ensure consistent annotation, a set of abstraction guidelines was developed for the annotators

Text-based clinical notes were evaluated, and key elements abstracted. A note was classified as containing a serious illness conversation if any one of the SIC domains were A-E were present. The same text could be used for multiple domains if deemed appropriate by the annotator.

Serious Illness Conversation Domain (SIC)

| **Code** | **Domain** | **Definition** | **Subthemes** | **Examples** |
| --- | --- | --- | --- | --- |
|  | Goals of Care - | Documented conversations with patients or family members about the patient’s goals, values, or priorities for treatment and outcomes. Includes statements that conversation occurred as well as listing specific goals. | 1. Documentation about overarching goals and patient values (General) | *"Patient stated that her goals were to be able to live long enough to go to her daughter’s wedding.”*  *“Hoping to continue to be aggressive in his cancer care.”*  *Negative Examples:*   - *“Feeling very well and enjoying an excellent quality of life.”* |
|  |  |  | 1. Documentation about discussion about preferences regarding EOL care or serious illness (General)   OR    Documentation that advance care planning was discussed, reviewed, or completed. | - *"We discussed what the patient would like as far as life-extending therapies if his condition should decline."* - *"Patient stated today that she would not want aggressive interventions if she were not able to interact with her family."* - *"I talked with patient about his values regarding end-of-life care. He believes that life is worth living as long as his heart is beating."* |
|  |  |  | 1. Documentation about discussion about patient preferences regarding cancer-directed therapy and how that fits in with their goals/values.   Any documentation about discussions with patient or surrogate about treatment options and how that fits in with their goals/values. This could include discussions surrounding the patient’s goals/values with regards to enrolling in a clinical trial, continuing with chemotherapy or surgery etc as long as it also includes discussion of goals and values. | - *“We discussed the options of pursuing further treatment with docetaxel, versus transitioning to best supportive care. Ultimately, we agree to move towards best supportive care, based on Jane’s goals and preferences.”* - *We discussed the pros and cons in the context of her priorities. She feels as if delaying starting therapy is not in line with her goals and values and she prefers to initiate therapy as soon as possible.”* - *“Hoping to continue to be aggressive in his cancer care.”*   Negative examples   - *“He is not against resuming chemo in the future if indicated.”* |
|  | Limitation of life-sustaining treatment | Documentation about preferences for limitations to cardiopulmonary resuscitation and intubation. | 1. Documentation that any of the following forms were discussed, given to the patient or surrogate, completed, or reviewed by the provider.  - Code status order - Documented Code Discussion - MOLST, Medical Order for Life Sustaining Treatment - Health Care Proxy or identification of surrogate decision maker - Living Will, Power of Attorney, Five Wishes, - Advanced Directive, Healthcare directive | For example:   - *“She is going to speak to her family about code status before signing a MOLST, but is leaning towards DNR/DNI”* - *“Jane reiterated her decision for a DNR/DNI CODE STATUS. Order entered into epic. MOLST is on file and she has a hardcopy present at her home.”* - *Documentation of SIC performed during the clinic visit* |
|  |  |  | 1. Documentation about discussion regarding specific patient preferences regarding advanced care planning or medical care as they approach the end of life | - *“She does not want to have prolonged post-intubation should she get COVID because she understand she still has advanced lung cancer to fight… She is leaning towards becoming DNR/DNI but wants to discuss more with family at this time.”* |
|  | Prognostic Awareness | Documentation about a discussion regarding estimation of chances for recovery (i.e., incurable disease or severe illness), limited time to live, estimation of shortened life expectancy, estimation of therapy goals (including risks of systemic chemo), or discussion about the course of disease.  *Prognostic understanding* refers to the perception of prognosis (ie, curability or life expectancy estimates). | 1. Documentations regarding patient’s understanding of prognosis | *“Patient illness understanding: Cancer is advanced but well controlled on EGFR TKI. Understands that age and underlying cancer make recovery from COVID19 a long and arduous process”* |
|  |  |  | 1. Documentation regarding physician’s perception of prognosis; | *“Prognositc information: Incurable shared”*  *“I am particularly concerned about the risks of systemic chemotherapy given her rapidly declining performance status and the current COVID-19 crisis. Therefore we agreed to continue best supportive care.”*  *“We discussed the covid pandemic and how she is at high risk of complications if she develops the disease due to steroids and lung malignancy. I therefore would have a high threshold to initiate any myelosuppressive systemic therapy in this period.”*  *“We also discussed the uncertainty regarding the severity of infection with COVID-19 should she initiate treatment with immunotherapy.”*  *“We discussed its not a good idea to pursue cancer directed therapy given current functional status.”* |
|  | Hospice | Documentation that hospice was discussed, prior enrollment in hospice, patient preferences regarding hospice, or assessments the patient did not meet hospice criteria. |  | Any documentation of discussions with patient or surrogate about hospice. For example:   - *“We discussed whether to pursue further hospice care versus consider restart systemic therapy. Both Jane and I agreed that further systemic therapy would likely be far more harmful than helpful.”*   Negative examples that do NOT represent discussions with patients or surrogates:   - "Plan: May consider hospice given no additional treatment options." |
|  | Palliative Care | Documentation that specialist palliative care was discussed, patient preferences regarding seeing palliative care clinician. |  | Any documentation about discussions with patient or surrogate about palliative care consultation or referral. For example:   - "Explained to patient what palliative care is and how they might help."   For an affirmative mark, the referral/consultation must be discussed with patient or surrogate.  Negative examples that do NOT represent discussions with patients or surrogates:   - *“He will benefit from early PC involvement.”* - *“Highly appreciate continued pall care follow up as well.”* |

Family/Proxy

| F | Family/Proxy | Documentation about communication with Family/HealthCare Proxy | 1. Family present with patient 2. Communication with family only 3. No family present |  |
| --- | --- | --- | --- | --- |

Cancer Status

| Code | Domain | **Definition** | **Subthemes** | **Examples** |
| --- | --- | --- | --- | --- |
| M | Cancer Status | Documentation about the status of the patient’s cancer at the time of conversation. | 1. Cancer: Progression: Documentation that the patient’s cancer is progressing, not responding to current therapy |  |
|  |  |  | 1. Stable or responding: Documentation that the patient’s cancer is stable, not progressing |  |
|  |  |  | 1. Cured/NED: Documentation that the patient’s cancer is not detectable, no evidence of disease |  |
|  |  |  | 1. New Diagnosis |  |
